# (B)On(e)-cohistones and the epigenetic alterations at the root of bone cancer

**DOI:** 10.1038/s41418-023-01227-9

**Published:** 2023-10-12

**Authors:** Paolo Salomoni, Adrienne M. Flanagan, Lucia Cottone

**Affiliations:** 1 Nuclear Function Group, German Center for Neurodegenerative Diseases (DZNE), 53127 Bonn, Germany; 2 Department of Histopathology, Royal National Orthopaedic Hospital, Stanmore, Middlesex HA7 4LP UK; 3 Department of Pathology, UCL Cancer Institute, University College London, London, WC1E 6BT UK

**Keywords:** Sarcoma, Epigenetics

## Abstract

Identification of mutations in histones in a number of human neoplasms and developmental syndromes represents the most compelling evidence to date for a causal role of epigenetic perturbations in human disease. In most cases, these mutations have gain of function properties that cause deviation from normal developmental processes leading to embryo defects and/or neoplastic transformation. These exciting discoveries represent a step-change in our understanding of the role of chromatin (dys)regulation in development and disease. However, the mechanisms of action of oncogenic histone mutations (oncohistones) remain only partially understood. Here, we critically assess existing literature on oncohistones focussing mainly on bone neoplasms. We show how it is possible to draw parallels with some of the cell-autonomous mechanisms of action described in paediatric brain cancer, although the functions of oncohistones in bone tumours remain under-investigated. In this respect, it is becoming clear that histone mutations targeting the same residues display, at least in part, tissue-specific oncogenic mechanisms. Furthermore, it is emerging that cancer cells carrying oncohistones can modify the surrounding microenvironment to support growth and/or alter differentiation trajectories. A better understanding of oncohistone function in different neoplasms provide potential for identification of signalling that could be targeted therapeutically. Finally, we discuss some of the main concepts and future directions in this research area, while also drawing possible connections and parallels with other cancer epigenetic mechanisms.

## Facts

- Oncohistones are the most compelling evidence for a causative role of epigenetic alterations in cancer.
- H3.3-G34 and H3.3-K36 mutations drive bone cancer by altering cell intrinsic (differentiation trajectories) and extrinsic (interaction with the tumour microenvironment) mechanisms.
- Oncohistones action in the bone shares mechanisms with brain-restricted oncohistones and other metabolic epigenetic alterations but also show unique features.
- A growing number of genetic alterations targeting epigenetic players have been implicated in other bone cancers and sarcomas and might inform on how oncohistones drive bone tumours.

## Open questions

- Investigation into mechanisms of action of oncohistones in the bone is warranted as it could be exploited for therapeutic interventions.
- Cooperation of oncohistones with other oncogenic signals has been suggested but requires full assessment.
- Mechanisms such as alterations of telomere biology, de-repression of retrotransposable elements, metabolic imbalance and induction of phase separation identified in other epigenetically-driven cancer may also contribute to oncohistone-driven tumourigenesis in the bone.

## The role of histone mutations in genome organisation in health and disease

### Epigenetic perturbations in cancer

Chromatin, the nucleoproteic complex composed of DNA, histones and non-histone proteins, plays a fundamental role in the regulation of DNA- and RNA-based processes ranging from DNA replication, DNA repair, maintenance of genomic stability and control of gene expression. Dynamic changes in chromatin structure and function ultimately orchestrate key biological functions such as cell cycle and cell fate determination, which have been covered previously in other reviews [1–3].

As a result of the increasing cancer sequencing studies, accumulating evidence in the past two decades has shown that virtually all classes of chromatin-associated epigenetic enzymes are frequently altered in cancer [4], suggesting that they play a driving role [5]. Compelling examples are mutations affecting the DNA methylation machineries, the chromatin remodelling complex Switch/Sucrose Nonfermenting (SWI/SNF) and the Polycomb Repressive Complex 2 (PRC2) complex (Box 1). The PRC2 complex can be targeted by both Loss of Function (LoF) and Gain of Function (GoF) mutations and more research is needed in this area to dissect their mechanisms of action and their consequences on cell and tissue physiological processes to define their causal relationship with cancer development.

Box 1 Common genetic alterations of the epigenetic machinery in cancer Mutations in the DNA methylation machineries were described at high frequency in clonal haematopoiesis [116] and haematological malignancies [117] and the identification of a role for Histone Acetyl transferases (HATs)/Histone Deacetylases (HDACs) in solid tumours has led to the first epigenetic drugs being tested in clinical trials [118].

The finding of deletions in the SWI/SNF Related Matrix Associated Actin Dependent Regulator Of Chromatin, Subfamily B, Member 1 (SMARCB1) gene in paediatric rhabdoid tumours has paved the way to the discovery that various components of SWI/SNF chromatin remodelling complexes, including Protein Polybromo-1 (PBRM1) and AT-Rich Interaction Domain 1 A (ARID1A), are among the most common mutations in human cancers [119, 120].

The SET domain methyltransferase Enhancer of Zeste Homologue 2 (EZH2), the core catalytic component of the PRC2 is frequently altered in cancer [121]. Activating GoF cancer-associated hotspot mutations and amplifications in *EZH2* have been reported in solid tumours and lymphomas in which histone hypermethylation has been linked to altered gene expression and transformation [122]. In contrast, LoF mutations are frequently seen in myeloid malignancies and leukaemias where they mediate neoplastic transformation [123]. The dual function of EZH2 in different tissues and cancers exemplifies the complexity of the role of epigenetic alterations in cancer. Some of these concepts have been discussed in a review by Bernstein and collaborators [124], whereby opening of chromatin following LoF of PRC2 could lower the epigenetic barrier to increase cell plasticity and aid the process of neoplastic transformation. In contrast, PRC2 GoF could increase the epigenetic barrier and lock transformed cells in a proliferative state and/or pro-survival mode. One could speculate that these mutations may occur at different stages of differentiation/development, where requirement for restrictive or permissive chromatin states for neoplastic transformation differs.

### Mutations in histone coding genes in cancer

The discovery of prevalent somatic mutations in specific paediatric brain cancer subtypes of histones, the highly conserved proteins that wrap ~147 bp of DNA around the nucleosome, the basic unit of chromatin (see Box 2), has provided key evidence for a causal role of epigenetic alterations in cancer. Seminal papers [6–9] reported recurrent driver mutations in the histone variant H3.3 and chromatin remodelling genes, paving the way to research in this area and leading to the identification of more histone mutations in several other cancer types [10] (expanded below). We and others refer to these mutations as ‘oncohistones’ to highlight their role in driving oncogenesis [11–13].

Non-synonymous substitutions in position 34 in histone H3.3, where a Glycine is changed for an Arginine or a Valine (G34R/V), were found in cortical paediatric high grade glioma (HGG), whereas Lysine to Methionine missense mutations at position 27 (K27M) in histones H3.3 (but also canonical H3.1/3.2 at a lower frequency) define paediatric diffuse intrinsic pontine gliomas (DIPGs)/diffuse midline gliomas (DMGs) [6–8].

A year after the reporting of brain cancer-associated H3.3 mutations, we identified recurrent somatic histone mutations in two rare benign bone tumours (covered in Section “Mechanisms of action of oncohistones in bone cancer”): Lysine 36-to Methionine or Isoleucine (H3.3-K36M or K36I) in chondroblastoma (CB) and Glycine 34-to Tryptophane or Leucine (H3.3-G34W or G34L) in giant cell tumour of the bone (GCT) [14], two locally aggressive benign cartilage and bone tumours, respectively.

Besides mutations in histone H3.3, several studies have highlighted that mutations in all core histones, including their globular domains, are found in various cancer types (covered in Box 3).

Box 2 Histones in chromatin regulation In the nucleosomes, the DNA is wrapped around an octamer consisting of two copies of histone core proteins (H2A, H2B, H3 and H4). These histones, referred to as canonical histones, are the most abundant components of the nucleosome and are synthesised in a replication-dependent fashion. However, a number of non-canonical histone variants, which differ by only a few amino acids from their canonical counterpart, are synthesised and incorporated into chromatin in a replication-independent manner, thus adding complexity to regulation of chromatin structure and function [125–128]. Furthermore, chemical groups are added as post-translational modifications (PTMs) to specific residues of the histones, more frequently at the N-terminal free tails: the type of chemical group together with the specific localisation represent a complex system devoted to decoding the epigenetic information into cellular processes, the ‘histone code’ [3].

Histone H3 variants, in particular, have come to the fore as important players in regulation of chromatin dynamics. The H3.3 variant (encoded by *H3-3A* and *H3-3B* genes) replaces the more common H3.1 (*H3C1*) and H3.2 (*H3C15*) both encoded by large gene clusters [126]. H3.3 marks enhancers of developmentally regulated genes where it is deposited by the Histone Regulator A (HIRA) complex. H3.3 incorporation into these genomic regions has been linked to recruitment of the PRC2 for trimethylation of Lysine 27 of the H3 tail (H3K27me3), a key repressive mark modulating gene repression during development [129]. In contrast, H3.3 is incorporated by the Death domain-associated protein (Daxx)–Alpha-thalassaemia X-linked mental retardation (Atrx) complex at centromeres, telomeres, retrotransposable elements (RTEs) and imprinted genes, where it mediates heterochromatin maintenance [130–134]. Daxx-dependent repression of RTEs has been reported as Atrx-dependent or -independent, and to rely on H3.3 incorporation or on Daxx/H3.3 interaction [132, 135]. Our work has shown that loss of Daxx leads to RTE de-repression and a marked myeloid bias during haematopoiesis, which ultimately results in induction of inflammation [72]. H3.3 incorporation and its PTMs have been linked to various nucleic acid-based processes including transcriptional regulation after DNA damage and in response to stimuli [136, 137] as well as activity-dependent regulation of transcription in neurons and neuronal plasticity with implications for behaviour [138–140]. Finally, H3.3 increases on chromatin during aging and has been implicated in regulation of the lifespan in nematodes [141, 142].

Box 3 Mutations in tails and globular domains of core histones Several mutations in all four core histones as well as in the linker histone (Histone 1, encoded by *H1-1* gene) [143] have been identified, some of which have been associated with specific cancer subtypes and their mechanisms of action at least partially elucidated [9, 10]. However, for most mutations, there is still no conclusive evidence for their role in tumourigenesis.

Cancer-associated mutations of histones H2B, H3.1 and H2A.Z.1 have been shown to alter the structure and the stability of nucleosomes [144]. H2B-E76K mutations are found in bladder and head and neck cancers, destabilise the core histone and deregulate chromatin structure and gene expression [144, 145]. Similarly, H2A-E57Q alterations (*HIST1H2AB* gene) are enriched in carcinomas of the female tract and increase the expression of EMT genes, migratory and invasive properties of cancer cells [146]. H2B-G53D is associated with pancreatic ductal carcinoma where it has been shown to increase gap closure ability and cell migration [147].

Besides mutations in the histone tail, a range of alterations in the globular domain have also been recently uncovered [9]. These affect transcription, dysregulate cell-signalling and cancer-associated gene pathways as well as impairing cellular differentiation in vitro [16].

## Mechanisms of action of oncohistones in bone cancer

**Fig. 1:**
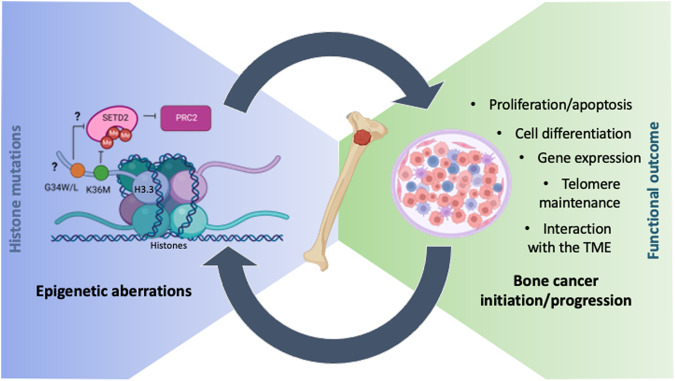
Overview of current understanding of the mechanisms of action of oncohistones in bone cancer. Causality of epigenetic aberrations in bone cancer initiation and progression including oncohistones and how the mechanobiology of histone mutations (left) correlates with the oncogenic functional outcome (right) is still an open area of exploration. Left: H3.3-K36M has a dominant negative inhibitory effect on the activity of SETD2, the histone methyltransferase responsible for H3K36 methylation, which is known to antagonize PRC2. H3.3-G34W/L mutants are suggested to block SETD2 binding, thus reducing its activity on neighbouring H3K36 methylation with consequent alterations of H3K27me and H3K27ac distribution: the evidence to support this model is mainly obtained from studies in brain cancer and it is conflicting in the literature. Mutations are orange (H3.3-G34W/L) and green (H3.3-K36M) circles; methyl groups (Me) are red circles.

The mechanism of actions of oncohistones are mutation- and tumour type-dependent. A plethora of evidence have contributed to the identification of the molecular events that drive oncogenesis in brain cancer (see Section “Oncohistones in brain and bone cancer: similarities and differences” and covered by ref. [15]). Most studies investigated post-translational modifications (PTMs) in the histone tails of both mutated and unmutated histones, but cancer-associated mutations in the globular domains were also shown to modulate chromatin remodelling events, histone exchange and nucleosome sliding, leading to cell lethality in yeast models [16]. Significantly less is understood of how oncohistones drive bone cancer: in this chapter, we will survey our current understanding of oncohistone’s mechanisms of action in the bone (Fig. 1).

### Oncohistone-driven chondroblastoma and giant cell tumour of the bone

Bone tumours and soft tissue malignant tumours form a class of tumours of mesenchymal origin called sarcomas that represent less than 2% of all human cancers. Mesenchymal neoplasms include at least 100 entities with diverse biological and clinical characteristics ranging from benign, locally aggressive, low-grade to highly aggressive tumours [17]. These display a variety of genomic alterations encompassing single point mutations, fusions, structural rearrangements and there are subsets particularly enriched in epigenetic aberrations [18].

Bone tumours, which account for about 13% of all sarcomas, arise in cells of bone or cartilage tissues, although the specific cell of origin is not known for most [19]. The remaining sarcomas arise in other tissues of mesenchymal origin such as muscle, fat and vascular structures [17]. Bone tumours can occur at any age but they are prevalent in children and young adults, and the most aggressive subtypes (high grade chondrosarcoma, Ewing sarcoma and osteosarcoma) are difficult to treat and generally have a poor prognosis [20].

GCT and CB are two benign but locally aggressive entities accounting for about 20% and 5% of all benign bone tumours, respectively, presenting with debilitating symptoms and with the potential to transform into high grade disease and metastasise in rare cases (see Table 1). GCT and CB are both characterised by highly recurrent and mutually exclusive single point mutations in histone genes, in the absence of other genetic alterations, supporting the idea that histone mutations play a major role in their oncogenesis [14, 21]. In GCTs, H3.3-G34 mutations are found only in the *H3-3A* gene and the vast majority are H3.3-G34W, although infrequently alternate H3.3-G34L/V/R/L alterations are detected in a subset of cases [14, 22, 23]. CBs harbour H3.3-K36M mutations in *H3-3A/B*, and more rarely K36I mutations restricted to the *H3-3B* gene [14, 24], suggesting that different expression pattern of H3.3 genes in specific cell types may impact the oncogenic role of these mutations (see Table 1).

**Table 1:** Overview of clinical and molecular features of oncohistone-driven bone cancers.

Bone cancer type	Clinical information	Putative tissue/cell of origin	Genetic alteration	Mutation frequency	Mechanism of epigenetic alteration	References
Chondroblastoma	- Predilection for children and adolescents (immature skeleton). - Epiphysis of long bones. - Malignant transformation in less than 1%.	Cartilage cells: chondroblasts	Histone H3 single point mutations K36M (*H3-3A/B*) and K36I (*H3-3B*).	95% of cases harbour K36M	Changes in chromatin regulation and gene expression	[14]
Giant cell tumour of bone	- Predilection for young adults (mature skeleton). - Epiphysis of long bones. - Malignant transformation in 5% of cases.	Bone cells: mononuclear stromal cells (immature osteoblasts)	Histone H3 single point mutations G34W/L/V/R (*H3-3A)*.	85-95% of cases harbour G34W	Changes in chromatin regulation and gene expression	[14]

It is intriguing that both tumours present in the epiphysis of bones in contrast to the metaphysis where most other bone tumours occur, and therefore interesting to speculate that both tumour types arise in the growth plate in the immature skeleton where the histone genes play a physiological role in bone development [25, 26].

### Mechanisms of action of K36 mutations in chondroblastoma

Most of the evidence around the mechanobiology of H3.3-K36M mutations comes from work performed by Lu et al. [27] and Fang et al. [28] who independently confirmed the oncogenic role of this mutation using in-vitro models. Mechanistically, K36M acts as a dominant negative inhibitor of SET Domain Containing 2 Histone Lysine Methyltransferase (SETD2), an enzyme that uniquely tri-methylates Lysine 36, and other nuclear receptor–binding SET Domain (NSD) family proteins due to an usual conformational structure of the histone interacting with the catalytic domain of SETD2 [29]. Expression of K36M-mutant histone in cellular models leads to widespread reduction of demethylation and trimethylation of Lysine 36 of the H3 tail (H3K36me2, H3K36me3) [30] but also affects the distribution of H3K27me3, a key repressive mark modulating gene repression during development [30, 31]. This is similar to how the K27M mutation acts, as it also impairs the deposition of opposing chromatin marks H3K27me3/2 and H3K36me3/2 [31].

How H3.3-K36M mutations promote their downstream effects in the specific context of cartilage, the tissue in which CB develop, has been explored in two seminal papers published in 2016. Here, the authors analysed K36M in disease-relevant models such as murine mesenchymal progenitors cells or immortalised human chondrocytes and found oncohistone-mediated global changes in gene expression, compromised differentiation, enhanced colony formation and impaired homologous recombination [27, 28]. Moreover, when transplanted in immunocompromised mice, only mesenchymal progenitors expressing the H3.3-K36M, but not the wild type (WT) counterpart, were able to develop into undifferentiated sarcomas [27], strongly making the case for this oncohistone being a driver for this disease.

The H3.3-K36M mutation also appears to modulate chondroblast biology via perturbation of cell differentiation trajectories: altering the H3K36me2/me3 landscape, the oncohistone increases the expression of genes linked to oncogenesis and augments colony formation capacity. This study also revealed that SRY-Box Transcription Factor 9 (SOX9) and Bone Morphogenetic Protein 2 (BMP2), critical regulators of chondrocyte differentiation, were among the downregulated genes [28].

### Mechanisms of action of G34 mutations in giant cell tumour of bone

Although most of the evidence behind our first understanding of how H3.3-G34 mutations impact cellular function comes from studies in brain cancer [15], recent work from us and others has started to clarify how they specifically work in the context of bone cells.

In contrast to K27 and K36 residues, position 34 of the H3.3 tail has to date not been reported as a PTM site, suggesting that G34 mutations exert their effects indirectly. Structural studies proposed that H3.3-G34 mutants inhibit SETD2 binding, thus reducing its activity on the neighbouring residue H3K36 methylation [29, 32]. H3.3-G34R/V/D activity *in cis* is associated with a significant decrease in H3K36me2/me3 on the mutant H3 tail and affects the function of SETD2 and Nuclear receptor binding SET Domain protein 1/2 (NSD1/NSD2), together with a reduced interaction with the mismatch-repair complex MutSα leading to a mutator phenotype [30, 32]. Jain et al. have confirmed, using mesenchymal stem cells as model, that the presence of G34 mutations directly alters the chromatin landscape at enhancers by obstructing methylation of H3K36 by SETD2 but not by the NSD1/2 enzymes. Consequently, this leads to aberrant gain of PRC2-deposited H3K27me2/3 and loss of lysine K27 acetylation (H3K27ac) at active sites, thus disrupting gene expression towards oncogenic programmes [33]. Furthermore, K36 mediates its effect differently to G34R/V/W mutants, which were found to reduce H3K36 methylation on the same histone (*in cis*) but not on neighbouring histones (in trans) showing that unlike K36 and K27, G34 oncohistones do not have a direct dominant-negative effect [29, 30, 34].

Lim et al. showed that the H3.3-G34W mutation promotes colony formation, infiltration and proliferation in primary GCT cell lines compared to GCTs that lack the mutations, as also recapitulated by H3.3-G34W knocked-in osteosarcoma MG63 cell line [35]. However, the effect of this mutation in the context of primary or at least immortalised cell lines remains to be investigated. Khazaei et al. used GCT patient-derived engineered cancer cells and patient-derived xenograft models to demonstrate that H3.3-G34W is necessary for tumour formation: reverting H3.3-G43W to WT significantly decreased proliferation, colony-forming ability in vitro as well as reduced growth when implanted in tibial orthotopic and subcutaneous models [36].

**Fig. 2:**
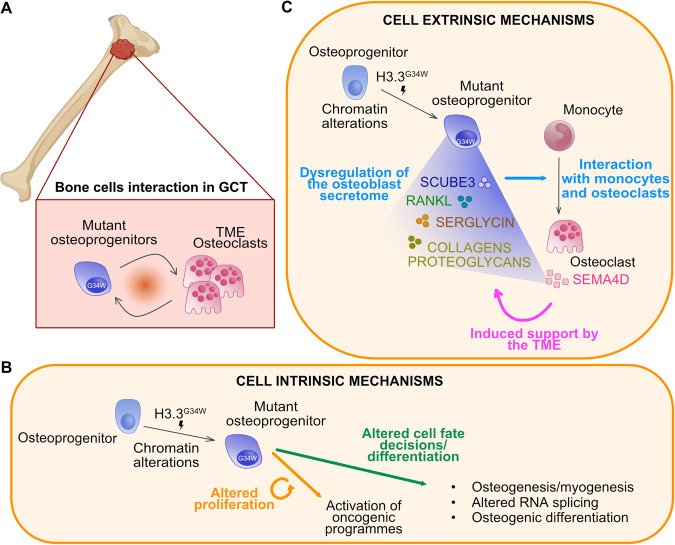
H3.3-G34W oncohistone drives GCT via multiple complex and multifaceted mechanisms of actions. **A** H3.3-G34W mutant osteoprogenitors in GCTs are in close proximity to and interact with giant multinucleated osteoclasts in the bone tumour microenvironment (TME). H3.3-G34W act via cell intrinsic mechanisms (**B**) and cell extrinsic mechanisms (**C**). **B** Mutant H3.3-G34W osteoprogenitors harbour an altered chromatin landscape that leads to activation of oncogenic programmes resulting in altered proliferation as well as modulation of cellular differentiation/fate pathways leading to aberrant cell fate/differentiation decisions. **C** Mutant H3.3-G34W osteoprogenitors show alterations in chromatin landscape leading to dysregulation of the progenitor’s secretome (RANKL, SCUBE3, SERGLYCIN, proteoglicans/collagens) with modified release of proteins. These secreted proteins interact with monocytes and osteoclasts in the TME, controlling their differentiation. Osteoclasts, in turn, secrete factors which support the growth of mutant osteoprogenitors.

Overall, the mechanism(s) of action of G34 appears significantly more complex compared to other oncohistones and have not yet been completely elucidated. The evidence reached so far points to broadly two major cellular programmes which are among the hallmarks of cancer [37]: control of cellular differentiation/fate and control of cancer cells interaction with the tumour microenvironment (TME) (Fig. 2). These are covered in the following paragraphs.

#### Effect on cellular differentiation

Oncohistones perturb the chromatin landscape, therefore, inducing cell intrinsic alterations in key cellular pathways [38]. Together with data showing that G34W mutations promote proliferation at least in some in-vitro and in-vivo models [35, 36, 39], multiple lines of evidence support the notion that H3.3-G34W also act by interfering with transcriptional programmes that control differentiation. When looking at H3.3-G34W mutant GCT-derived primary cells, Lutsik et al. identified impaired osteogenic differentiation concomitant with global epigenetic alterations particularly at heterochromatic and bivalent sites [40]. Lim et al. also highlighted that the mutant histone interacts specifically with RNA components related to splicing and alters splicing events leading to aberrant transcription in GCT-derived primary cell lines [35]. Moreover, immortalised GCT cells engineered to lose and re-gain H3.3-G34W mutant were found to downregulate genes involved in muscle functions, suggesting that the mutations impair differentiation in a mesenchymal progenitor committed to myogenesis rendering mutant stromal cells as osteoblast progenitors [36]. In further support of this notion, we have recently demonstrated, using a human osteoblastic cell line, that overexpression of H3.3-G34W strongly impaired osteogenic differentiation but did not alter proliferation, migration or survival [41].

#### Effects on the tumour microenvironment

Thanks to the seminal discoveries in the last two decades, it is now clear that together with cell intrinsic, also cell extrinsic mechanisms in which cancer cells cooperate with components of the TME, such as stromal and immune cells, are critical for tumourigenesis [42]. Importantly, it has emerged that the TME can be exploited for cancer treatment, in particular with immunotherapy [43]. Tumour initiation and progression can also be sustained in a paracrine manner, as exemplified by the rare tumour craniopharyngioma in which Beta-Catenin-mutant cells form clusters of senescent cells that release Senescence Associated Secretory Phenotype (SASP) factors to stimulate neighbouring unmutated cells to proliferate as a potential non-cell autonomous cancer mechanism [44].

Our recent work and that of others support the notion that GCT should be considered a disease of aberrant paracrine signalling between mutant osteoprogenitors and the TME. GCT comprises mutant osteoblasts/osteoprogenitors, which are interspersed with multinucleated osteoclasts (derived from myeloid precursors) characterised by an abnormally elevated number of nuclei [45]. Osteoclast formation is dependent on Receptor activator of nuclear factor kappa-Β ligand (RANKL) and the most compelling evidence for a role of the TME in GCT pathogenesis is the clinical observation that Denosumab, a humanised antibody to RANKL, which blocks osteoclast formation, controls tumour growth by reducing mutant osteoblast proliferation and increasing maturation resulting in more bone formation [41, 46]. Recent work has shown that knock down of H3.3-G34W induces a reduction of *RANKL* mRNA expression by 57% [39] and that increased expression of *RANKL* in mutant GCT primary cells is associated with the presence of active histone marks at the gene regulatory sites [40]. However, mechanistic evidence for a role of RANKL in GCT pathogenesis is still lacking [47]. Furthermore, RANKL is not the only secreted factor altered in bone by oncohistones, as recently shown by Khazaei et al. [36]. Profiling of the secretome of isogenic immortalised GCT cell lines revealed that extracellular matrix (ECM) ligands such as collagens (Collagen Type VI Alpha chain 1/3, COL6A1/3 and Collagen Type V Alpha chain 2, COL5A2) and proteoglycans (Biglycan, BGN), which are predicted to interact with integrin receptors on monocyte/macrophages (Integrin Subunit Beta 2, *ITGB2*) and osteoclasts (Integrin Subunit Alpha V, *ITGAV*) respectively, were differentially secreted by H3.3-G34 mutant cells.

Our recent work showed that H3.3-G34W reduced transcription of several osteoblast-derived secreted factors including Signal peptide CUB Domain and EGF-like domain containing 3 (*SCUBE3)*, a Transforming Growth Factor (TGF)-beta family member which physiologically constrains formation of osteoclasts via monocytes fusion. Proliferation of mutant cancer cells is sustained primarily by their cross-talk with giant osteoclasts which secrete unregulated amount of Semaphorin D providing a growth advantage [41]. Similarly to SCUBE3, the proteoglycan Serglycin has been shown to be secreted by neoplastic GCT osteoblasts to promote osteoclast differentiation, contributing to a self-sustaining loop for tumour growth [48].

## Oncohistones in brain and bone cancer: similarities and differences

A great deal of the research on oncohistones has been conducted to identify their role in brain tumourigenesis, and the findings of their role in bone cancer has highlighted how the same mutations in different tissue types share some mechanisms of action but also show unique features. In this chapter, we highlight the similarities and differences between oncohistone-driven mechanisms of actions in brain and bone cancer.

One of the first studies which investigated how H3.3-G34V drive HGG identified differential binding of H3K36me3 at regulatory regions of transcriptional regulators involved in neuronal differentiation and cellular proliferation including *MYCN* [49]. G34R/V were also shown to alter H3K36me2 as they reduce access of the Lysine (K)-Specific Demethylase 2 (KDM2A) [50]. Voon et al. modelled H3.3-G34R targeting in mouse embryonic stem cells and demonstrated that it causes widespread changes not only in H3K36me3 but also in H3K9me3 as it interferes with the KDM4 family, lysine demethylases of lysines K9/K36 [51]. Similarly to the effect in the brain, H3K36me2/3 distribution is also affected by H3.3-G34W mutants in GCT [36, 41] (see Section “Mechanisms of action of G34 mutations in giant cell tumour of bone”). H3.3-G34W mutations have been shown to lead to alteration in global DNA methylation in GCT [40], similarly to HGG where G34W mutant tumours also show widespread DNA hypomethylation particularly at subtelomeric regions [52]. G34R also restricts the deposition of DNA methyltransferase 3 A (DNMT3A) and causes DNA hypomethylation, together with H3K36me2 alterations and epigenetic reprogramming, in the context of H3.3-G34R/V/W-associated neurodevelopmental disorders [53] (see also Section “Final remarks and future directions”). The mode of action of G34R and G34W has also, however, been suggested to be different, as they may diversely affect H3K36 modifications, subtelomeric silencing and genomic stability, as suggested in model yeast [54].

A significantly different mechanism compared to the H3.3-G34 mutations is described for the H3-K27M mutation, which is found in the brain where it acts as GoF to inhibit EZH2. While EZH2 is a frequent site of LoF and GoF mutations (see Box 1), H3-K27M in DIPG inhibits EZH2 enzymatic activity, leading to global reduction of H3K27me3 levels and affecting other major chromatin marks [7, 30, 52]. The model of PRC2 regulation by H3-K27M is not fully clear and multiple mechanisms of action have been proposed e.g., see ref. [55]. Moreover, inhibition of H3K27me3 is achieved in several cancers by interfering with multiple pathways upstream of this histone mark. Recently, Jain et al. demonstrated that molecular mimicry is used by EZH Inhibitory Protein (EZHIP), a molecule normally expressed in the placenta and uniquely upregulated in posterior fossa type A ependymomas where, similarly to H3-K27M, impedes spreading of H3K27me3 by interacting with allosterically activated PRC2 [56].

The mechanisms of action of the brain-restricted H3-K27M mutations also contrasts with the CB-restricted H3-K36 mutations, as these mutations affect the distribution and localisation of the opposing H3K27me3/2 and H3K36me3/2 chromatin marks (see Section “Mechanisms of action of K36 mutations in chondroblastoma”).

### Effects on cellular differentiation

With respect to the effect on cellular differentiation, significant overlap exists between bone and brain tumours (see also Chapter 2.3.1). In HGG H3.3-K27M/L regulate the expression of genes involved in neuronal differentiation in normal embryonic stem cells, and this appears to occur in a PRC2-independent manner [57]. Similarly, H3.3-G34R/V-bearing gliomas were shown to be neuronal malignancies in which interneuron progenitors are stalled in their differentiation trajectory by the oncohistone, and co-option of platelet-derived growth factor-alpha receptor (PDGFRA) signalling further promotes malignant transformation which is associated with astrocytic features [58]. It is presently unknown whether the signalling of PDGFRA, which was reported to be expressed but not mutated in GCT [59], is involved also in pathogenesis of this disease.

Interestingly, H3.3-K27M and H3.3-G34R show a strong predilection for occurring in tumours at distinct anatomical sites, due to mechanisms not entirely clarified. Bressan et al. recently demonstrated that cell-intrinsic regional identity provides differential responsiveness to different oncohistones [60]. Moreover, H3.1- or H3.3-K27M mutations occur in distinct oligodendroglial cell lineages in brain midline gliomas [61]. This ties together with the evidence that stalling of developmental programmes seems to be a feature shared by several paediatric gliomas [62].

The action on cellular differentiation extends also to globular mutations. Recent evidence by Bagert et al. have shown that cancer-associated histone mutations in the globular domain affect nucleosome stability (H2B-E71K/Q and H2B-E76K/Q) and nucleosome sliding (H2B-E113K/Q) and profoundly affects cell fate decision and differentiation of murine mesenchymal progenitor cells by altering gene expression of pathways involved in cell adhesion and pluripotency [16]. It remains to be determined whether the same mechanism applies to other less common histone core mutations.

### Effects on interaction with the tumour microenvironment

Work by Haase et al. has also uncovered that G34 mutations may contribute to HGG by altering interaction with the immune system (see also Section “Effects on the tumour microenvironment”). Using a syngeneic genetically engineered mouse model of H3.3-G34R-driven HGG the authors demonstrate that this oncohistone induces down-regulation of DNA repair pathway. In turn, this causes genetic instability and accumulation of extrachromosomal DNA. Ultimately, the latter activates the cyclic GMP–AMP synthase/stimulator of IFN genes (cGAS/STING) pathway, inducing the release of immune-stimulatory cytokines [63].

### Possible parallels: H3.3 chaperones and telomere maintenance

One possible secondary effect of the presence of oncohistones that has emerged from studies on HGG, revolves around the role of Histone H3.3 at telomeric regions. H3.3 is incorporated at telomeric regions coupled to histone H4 in nucleosomes exquisitely by Alpha-thalassaemia X-linked mental retardation (Atrx); Death domain-associated protein (Daxx) can also contribute to H3.3 deposition at telomeres recruiting a pool of non-nucleosomal H3.3 to Promyelocytic Leukaemia nuclear bodies prior to deposition [64]. H3.3-G34R/V mutations found in cortical HGG (H3.3-G34R found in 20% of cases, H3.3-G34V less commonly) are frequently coupled to alterations in *ATRX/DAXX* and *Tumour Protein 53* (*TP53)* genes [6] leading to induction of the alternative lengthening of telomeres (ALT) phenotype, whereas this association is never found in GCT samples [14, 21].

There is some evidence that G34R might trigger ALT irrespective of the Atrx status in the brain [6, 65], making it tempting to speculate that also H3.3-G34W might influence telomere lengthening in the absence of Atrx mutations [14]. Lutsik et al. showed that H3.3-G34W GCT primary cells display non-recurrent centromeric fusions, which could be a potential consequence of heterochromatin defects induced by the oncohistone and could potentially contribute to genomic instability [40]. Telomere dysfunction has been implicated in malignant transformation of benign GCT to malignancy: malignant H3.3-mutated tumours are enriched for a variety of alterations in Telomerase Reverse Transcriptase (TERT) [21]. Forsyth had previously shown that TERT-dependent telomere maintenance, and not ALT, is possibly playing a major role in GCT [66]. It is worth noting that Atrx loss associated with ALT activation are frequent events in multiple sarcomas [67]. Interestingly, most high grade osteosarcomas activate ALT either in the absence of Atrx, with a more aggressive tumour cell phenotype and ECM remodelling [68], or in the presence of Atrx in conjuction with an amplification of the ALT-regulator TOP3A [69]. Finally, mutations inhibiting KDM4B drive ALT activation in Atrx-mutated glioblastomas, suggesting cooperation between these genetic alterations in promoting ALT [70].

More work needs to be put into dissecting if and how alterations in telomere maintenance play a role in the pathogenesis on bone tumours, including GCT where Atrx alterations are not found genetically coupled with H3.3-G34W. In this respect, it is possible that additional mechanisms for ALT activation are implicated in GCT and should be further explored.

### Possible parallels: linking retrotransposable elements and oncohistones?

The transcriptional consequences of oncohistones are overall still incompletely understood, and the changes observed in the model systems studies might be the result of compensatory mechanisms more than direct effects of the mutations. In support of this hypothesis, it has emerged that the effect of K-to-M mutations in the brain might be explained by far more complex epigenetic mechanisms, such as the pervasive global re-distribution of H3K27ac marks resulting in the baseline increased expression of silent repetitive regions including retrotrasposable elements (for instance, endogenous retroviruses, ERVs). This observation is relevant to treatment of HGG, as H3-K27M cells are vulnerable to treatment with epigenetic inhibitors (DNA methylation and HDAC inhibitors) that further enhance the expression of repetitive elements and selectively stimulate an endogenous antiviral response [71]. Recently, de-repression of ERVs has been demonstrated also in models of Drosophila Melanogaster by K27M and K36M mutations, which induce developmental defects caused by redistribution of antagonistic marks H3K27me3 and H3K36me2: H3K36me2 is lost in pericentric heterochromatin leading to de-repression of ERVs [31]. Interestingly, loss of Daxx or Atrx (covered also above), which is found in multiple human cancers from paediatric HGG to osteosarcoma to myeloid malignancies, has been reported by us and others to lead to de-repression of ERVs [72–75]. Whether deregulation of ERVs is a consequence of other bone-restricted oncohistones such as G34W/L and whether it plays a role in sarcomagenesis is still an open question.

## Epigenetic roots of bone cancer and sarcomas beyond oncohistones

**Fig. 3:**
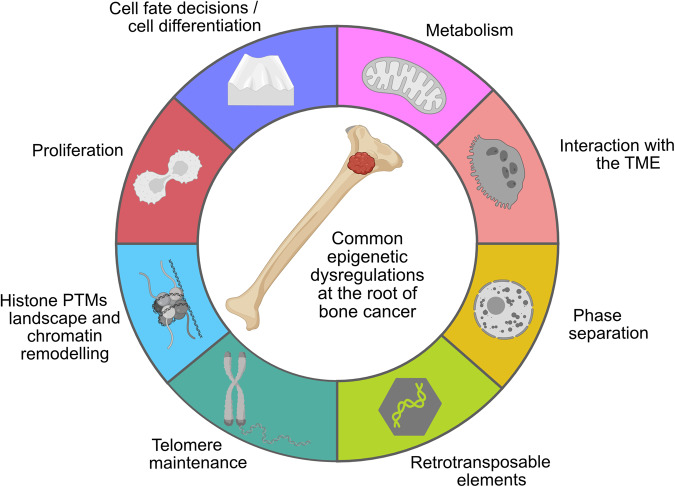
Common intrinsic and extrinsic epigenetic dysregulations that have been uncovered so far at the root of bone cancer. Histone post-translational modifications (PTMs) landscape and chromatin remodelling (common to all epigenetically driven sarcomas); proliferation (i.e., oncohistone- and IDH-driven tumours); cell fate decision/differentiation of the cell of origin (i.e., osteoblasts and chondroblasts by oncohistone, chondroblasts by IDH); metabolism (2-HG in IDH mutated tumours); interaction with the TME (i.e., osteoblast-osteoclasts interaction in oncohistone-driven GCT); phase separation (i.e., fusion proteins in various sarcomas); retrotransposable elements (possibly at the basis of bone cancer, still to be uncovered); telomere maintenance (i.e., possible atrx/daxx-independent telomere lengthening in GCT). Created with BioRender.com.

Besides oncohistones, a plethora of genetic alterations targeting several other epigenetic players have been implicated in bone cancers and sarcomas more generally (see also [18] and Fig. 3). This chapter will review some of the most recent findings on the epigenetic roots of bone cancer.

### Metabolic reprogramming and the epigenome in IDH-driven tumours

The majority of chondrosarcomas, the most common primary malignant bone tumour of adults, harbour single point mutations in the cytosolic *isocitrate dehydrogenase type 1* (*IDH1*; 60% cases) and in the mitochondrial *isocitrate dehydrogenase type 2* (*IDH2*) (10% cases) [76, 77], key enzymes in the tricarboxylic acid cycle [78]. Mutant IDH1/IDH2 acquire a neomorphic enzymatic function leading to the accumulation of the 2-D-hydroxyglutarate metabolite (2-HG) metabolite. 2-HG leads to global DNA hypermethylation, as also shown in astrocytomas and haematopoietic malignancies carrying IDH mutations [79, 80]. IDH mutations are also prevalent in more than 20 other tumour types including a subset of HGG (80% for low-grade and secondary glioblastoma), acute myeloid leukaemia (20%) and cholangiocarcinoma (20%) [79].

A plethora of work has reported that IDH mutations promote metabolic adaptations [81–83], altered cellular differentiation [84, 85] and metabolic reprogramming of the immune cells infiltrating the tumour [86, 87]. How much these mechanisms overlap with those at the basis of oncohistones action has not been determined yet. Similarly to the action of oncohistones, alterations of cellular differentiation appear to be the mode of action of IDH mutations, which impair histone and DNA methylation preventing lineage-specific progenitors to terminally differentiate [84, 85, 88]. IDH1 R132C and IDH2 R172K are associated with increased global histone methylation leading to increased dysregulation of glial differentiation [85], increased chondrogenic differentiation of human mesenchymal stem cells (IDH1 R132C, [84]) and reduced adipogenic differentiation respectively (IDH2 R172K, [85]).

Furthermore, the importance of the interaction with the TME has been demonstrated also for IDH-mutated tumours, where IDH-mutant cancer cells instruct macrophages to develop along an immunosuppressive phenotype by modulating tryptophan metabolism [87].

Interestingly, a recent study has reported that IDH mutations and H3.3-K27M modulate the Krebs cycle in an opposite fashion to sustain hypermethylation and hypomethylation, respectively [89]. What remains to be investigated is whether the widespread chromatin opening in K27M tumours is followed by compensatory mechanisms for chromatin closing that could rely on metabolic changes promoting an increase in repressive histone marks. In this respect, it is known that certain loci become repressed via H3K27me3 in DIPG despite the presence of H3.3-K27M [90].

### Epigenetic alterations identified at low frequency in bone cancers

Besides IDH mutations, a significant number of alterations in epigenetic regulators are identified in bone cancers at lower frequencies, making it difficult to establish a causative link in cancer pathogenesis.

Osteosarcoma, the most common bone cancer in children, is characterised by a highly disrupted genome with alterations of several tumour suppressor genes (mainly *TP53*, Retinoblastoma, *RB1*) and oncogenes. A proportion of osteosarcoma harbours alterations in Atrx (see above), associated with a more aggressive behaviour with increased growth and migration [68]. Loss-of-function of PRC2 is also frequently found in osteosarcoma cell lines but it is rarer in tumour samples [91]. Moreover, the H3.3-G34R/V substitutions, which do not occur in GCT, are instead found in osteosarcomas at low frequency (1–14%) [6, 7, 14].

Although at the genetic level GCTs are characterised exclusively by histone mutations, their malignant counterparts show the acquisition of further aberrations including the biallelic loss of KDM4B or KDM5A, two epigenetic enzymes that demethylate lysines of both active and repressive marks, suggesting an epigenetic rewiring of cancer cells during malignant transformation [21]. Similarly, a subtype of chordomas (poorly differentiated subtype), a rare tumour of the spine and base of skull, is associated with loss/inactivation of SWI/SNF Related Matrix Associated Actin Dependent Regulator Of Chromatin, Subfamily B, Member 1 (SMARCB1) [92]. Hotspot mutations in *H3-3A* have been reported in a proportion of high grade bone tumours, some with complex copy number aberrations and a DNA methylation profile more similar to that of osteosarcomas; these may represent transformation of GCT to high grade disease [93] and these findings reflect the difficulty in tumour classification.

### Further epigenetic players altered in sarcomas

Alterations in histones and other epigenetic modifiers are also described with various degree of frequency in a wide variety of sarcomas: understanding their mechanisms of actions may also inform on oncohistone biology, making this an exciting area of research.

The H3.3 variant is overexpressed in alveolar rhabdomyosarcoma, one of the most common sarcomas in children, where it increases the migratory and metastatic potential of cancer cells in a Melanoma Cell Adhesion Molecule (MCAM)-dependent manner [94]. K36M alterations are also found rarely in paediatric soft tissue sarcomas although they preferentially target H3.1 [23]. Some undifferentiated round cell sarcomas, a group of highly aggressive mesenchymal tumours that affect children and young adults, harbour mutations in the histone H1 (*HIST1H*) family members and show loss of SMARCB1 [95].

The histone ‘writers’ Suppressor of Zeste 12 (SUZ12) and Embryonic Ectoderm Development (EED), components of PRC, are frequently and mutually exclusively altered by LoF in sporadic cases of malignant peripheral nerve sheath tumour (MPNST), a rare sarcoma arising within peripheral nerves, generally in the presence of driver mutation in Neurofibromatosis type 1 (NF1) [96]. Members of the SWI/SNF remodelling complex are also altered in MPNST as well as in other sarcomas such as poorly differentiated chordoma, malignant rhabdoid tumours and epithelioid sarcoma [18].

Many sarcomas are driven by fusion proteins, which most commonly include FUsion in malignant lypoSarcoma (FUS), EWS RNA Binding Protein 1 (EWSR1) and TATA-box binding protein Associated Factor 15 (TAF15) as partners. FUS, EWSR1 and TAF15 are major drivers of a novel recently described mechanism of epigenetic regulation at the level of chromatin 3D structure, called phase separation. Phase separation refers to the phenomenon in which a supersaturated solution of components, as it is the case of biomolecules in chromatin, separates in two distinct independent and stable phases leading to membraneless compartimentalisation of biological processes, also called condensates [97]. There is supporting evidence that several transcription factors, such as the 30 members of the FUS family [98], use liquid-liquid phase separation and that formation of condensates may affect pathogenesis of neurodegenerative diseases and cancer [97, 99]. In Ewing sarcoma, the second most common bone cancer affecting children and young adults, the fusion involves EWSR1 and friend leukaemia virus integration 1 gene (EWSR1:FLI1) in nearly 85% of cases, alongside EWSR1:ERG in 10% of cases and rarer fusions with other members of the E-twenty-six (ETS) family of transcription factors). EWSR1:FLI1 targets critical chromatin regulatory complexes such as the SWI/SNF remodelling complexes via phase separation to establish and maintain oncogenic gene expression programmes at high levels to drive cancer [100–102]. Other epigenetic components that are involved in sarcomagenesis and other cancers may do so by causing phase separation [103]. PRC2 deposits H3K27me3, which in turn attracts PRC1 containing Chromobox (CBX) proteins: CBX2 undergoes phase separation to form condensates and organise polycomb-bound chromatin [104, 105]. One could speculate that oncohistones could cause aberrant partitioning of epigenetic enzymes similarly to how fusion proteins generate multimeric associations via their intrinsically disordered prion-like regions [18], and that phase separation might be one of their mechanisms of action.

Finally, an oncogenic fusion has been reported in Endometrial Stromal Sarcoma (ESS), where the oncohistone-mimic EZHIP is fused to the member of remodelling complex MBT Domain containing 1 (MBTD1) (MBTD1:EZHIP) [106]: it is therefore intriguing to speculate that impaired K27me3 spreading is also at the basis of this sarcoma. Intriguingly, Ragazzini et al. have shown that while the EZHIP transcript is undetectable in most osteosarcoma cell lines, it is expressed in the cell line U2OS leading to the possibility that EZHIP may play a role also in this bone cancer [107].

## Final remarks and future directions

The discovery of oncohistones have revolutionised the field of cancer epigenetics but their functions and mechanisms of action remain still an area of active exploration. Their roles in modifying the TME for supporting tumour growth and/or blocking of differentiation warrant urgent investigation, as they could be exploited for therapeutic intervention [108].

Another question is the degree of overlap with respect to downstream effectors of oncohistones compared to other epigenetic regulators, such as IDH. Oncohistones could co-operate with other oncogenic events like structural variants, as recently shown for paediatric high grade gliomas [109], or whole genome genomic aberrations in specific driver combinations during bone cancer progression.

Moreover, not only the location and cell of origin but also the timing at which these epigenetic alterations occur is likely critical for their mode of action. In this respect, mutations arising during development or postnatally may lead to unique behaviours although the downstream effectors might be common.

It has recently emerged that dysregulation of histones and their modifications leads not only to cancer, but it has also been associated with neurodevelopmental syndromes, psychiatric disorders and cardiovascular disease. Germline mutations in epigenetic modifiers cause neurodevelopmental syndromes with a wide spectrum of phenotypes, raising the possibility that the cell types in which such mutations occur and/or mutated gene dosage and timing may play key roles [110, 111]. Mouse models of H3.3 knock outs implicate also histone genes in developmental dysregulation [112]. Bryant et al have recently screened a large cohort of families bearing de-novo germline alterations in *H3-3A* and *H3-3B* genes and displaying neurodegenerative disorders without malignancies [113]. Moreover, germline H3.3-G34R/V mutations cause severe neurodevelopmental defects by decreasing H3K36me3 on H3.3 mutant tail, similarly to its cancer-associated counterpart, impairing recruitment and distribution of DNMT3A and ultimately silencing neuronal genes and activating neuroinflammatory pathways [53] (see also Section “Oncohistones in brain and bone cancer: similarities and differences”). Germline alterations are also found in the histone H4 and have been shown to induce genome instability and modulating cell cycle during early development resulting in neurodevelopmental phenotypes [114]. Interestingly, these syndromes are not associated with susceptibility to cancer, suggesting that their impact on early development is different from the one on more subsequent stages, where it leads to neoplastic transformation. Based on the identification of histone mutations in neurogenerative developmental disorders, it is tempting to speculate that selected orphan bone syndromes could also be caused by histone mutations or other epigenetic alterations, such as Ollier disease/Maffucci Syndrome caused by IDH1/2 mutations [78]. Supporting this idea, H3.3-G34W mutations have been detected also in a newly described cancer syndrome, where the mutation is thought to arise postzygotically, involving pheochromocytomas and paragangliomas together with GCT [115]. Understanding how histone mutations cause disease will provide the key to understanding further how epigenetic alterations in the downstream networks lead to neoplastic and non neoplastic disease and how they can be targeted for patients’ benefit.

## Supplementary information

Reproducibility checklist
